# Vestibulo-Ocular Reflex Is Modulated by Noisy Galvanic Vestibular Stimulation

**DOI:** 10.3389/fneur.2022.826739

**Published:** 2022-02-17

**Authors:** Akiyoshi Matsugi, Tomoyuki Shiozaki, Hiroaki Tanaka

**Affiliations:** ^1^Faculty of Rehabilitation, Shijonawate Gakuen University, Daitou, Japan; ^2^Department of Otolaryngology-Head and Neck Surgery, Nara Medical University, Kashihara, Japan; ^3^Department of Physical Medicine and Rehabilitation, Kansai Medical University, Hirakata, Japan

**Keywords:** noise stimulation, galvanic vestibular stimulation, video head impulse test, vestibulo-ocular reflex, postural control

## Abstract

We investigated whether noisy galvanic vestibular stimulation (nGVS) modulates the vestibulo-ocular reflex (VOR) and whether this effect is correlated with the effect of nGVS on body sway. Thirty healthy young adults participated. The video head impulse test (vHIT) was used to estimate the ratio of eye motion velocity/head motion velocity to VOR-gain. The gain 60 ms after the start of head motion (VOR-gain-60 ms) and regression slope (RS) (i.e., gain in eye and head motion; VOR-gain-RS) were calculated. The total path length of the foot center of pressure (COP-TL) during upright standing was calculated to estimate body sway. Noisy Galvanic Vestibular Stimulation at 0.2, 0.6, 1.2 mA, or sham stimulation (direct current: 0 mA) was delivered to the bilateral mastoid process in random order during vHIT and COP measurements. Application of nGVS at 0.2 mA significantly reduced VOR-gain-RS, while application of nGVS at 0.6 mA significantly increased COP-TL. Vestibulo-ocular reflex-gain-60 ms differed significantly between 0.2 and 1.2 mA. There was no significant correlation between COP-TL and VOR-related parameters. These findings suggest that nGVS at 0.2 mA inhibits the VOR, while nGVS at 0.6 mA increases body sway during upright standing, although there may be no relationship between the respective effects in healthy individuals.

## Introduction

The vestibulo-ocular reflex (VOR) is important for dynamic gazing in daily living (1), and patients with vestibular disease experience impairments in the VOR and dynamic visual ability (2). During the VOR, endolymph moves in the opposite direction of head movement, causing deflection of the ampulla (3), which in turn generates afferent action potentials in the primary vestibular nerve. In response to this impulse, the vestibular nucleus generates an action potential in the external eye muscles, resulting in eye movement. Thus, accurate assessment of VOR function is important for vestibular rehabilitation.

The head impulse test (HIT) is one of the most useful techniques for determining vestibular hypofunction and related vestibular disorders, and uses an impulsive VOR method first described by Halmagyi and Curthoys in 1988 (4, 5). Further, this HIT was implemented concomitant to the video discovered by Hamish McDougall (6), and this video head impulse test (vHIT) was compared with the gold standard search coil to highlight the saccades behavior in cases of chronic vestibular deficit. Further, the vHIT test specifically explores Type I Hair Cells activity and consequently afferent transient systems (5).

The ratio of the velocity of eye movement to the velocity of head movement is considered the gain of the reflex and is used to evaluate VOR functionality (7). In patients with unilateral vestibular disorder, the VOR gain is reduced by approximately 30% compared to the intact side (5). Furthermore, the vHIT can detect a slight change in VOR gain (8) following intratympanic gentamicin treatment in patients with Ménière's disease (9). Therefore, restoration of VOR-gain in patients with vestibular disorders has become an important issue in vestibular rehabilitation.

Galvanic vestibular stimulation (GVS) is used to change the excitability of the vestibular reflex as a non-invasive neuromodulation method, and has been shown to improve vestibular rehabilitation results (10). Galvanic vestibular stimulation can induce impulses in primary otolithic neurons as well as primary semicircular canal neurons (11, 12). Recent studies have reported that noisy galvanic vestibular stimulation (nGVS) has the potential to alter the excitability of the vestibular reflex (13–15) via the application of a noise current to the bilateral mastoid process. A possible mechanism for this alteration is interference by nGVS in vestibular information carried by the irregular vestibular neurons originating from Type I Hair Cells (16). Although nGVS is thought to alter the degree of vestibulospinal reflexes and body sway in both patients with vestibular disease (17) and healthy participants (18, 19), it remains unclear whether nGVS alters the VOR. In the present study, therefore, we investigated whether nGVS induces changes in VOR-gain.

The effect of nGVS on body sway is dependent on the stimulus intensity; if it is too weak, it has no effect, and if it is too strong, it may increase the vestibulospinal reflex. This dependence may also be true for VOR. A previous study reported that the optimal stimulus intensity was approximately 0.2 mA (20), with exacerbation occurring at approximately 0.5 and 1 mA. Therefore, in this study, we also examined the hypothesis that VOR-gain would be increased at 0.2 mA and decreased at 0.6 and 1.2 mA.

The effect of nGVS varies among individuals (18, 20). Even at the same intensity, the center of gravity sway may decrease or increase. If the effect of nGVS on gravity oscillation and the effect on VOR are caused by the same effect of electricity on the vestibular apparatus, then the amount of both effects should be correlated. Therefore, the present study further examined whether there is a correlation between the center-of-gravity sway caused by nGVS and the gain of VOR.

## Methods

### Participants

The appropriate sample size for one-sample tests and one-way repeated-measures analysis of variance (ANOVA) was estimated using G^*^power software (Version 3.1.9.4) (21) before the experiments. For one-sample tests, an *a priori* power analysis with the effect size (*d*) set to 0.8, the alpha error probability set to 0.05, and the power (1 – beta error probability) set to 0.95 indicated a required sample size of 23. For one-way repeated-measures ANOVA (OR-ANOVA), an *a priori* power analysis with the effect size (*f*) set to 0.4, alpha error probability set to 0.05, power (1 – beta error probability) set to 0.95, correlation among repetitive measures set to 0.5, and non-sphericity correction epsilon set to 1 indicated a required sample size of 15.

Thirty healthy adults (mean age, 20.5 ± 4 years; 23 women and 7 men) participated in the present examination. No participants had a history of neurological disease, including epilepsy, and none had experienced vertigo or dizziness within 3 weeks before participation in the study. All procedures of the present study were approved by the Ethics Committee of Shijonawate Gakuen University (approval code: 21-7) and were conducted with the understanding and written consent of each participant in accordance with the principles and guidelines of the Declaration of Helsinki.

### General Procedures

This study was conducted using a single-blind, sham-controlled design. The stimulation conditions of nGVS were not known to the participants or assessors for vHIT. Before examination, we tested inducing body sway to the anodal side (22) by a 2.5-mA square wave pulse (spGVS) (23, 24) with 200 ms duration (25, 26) while participants maintained upright standing with their head facing forward (26), eyes closed, and feet together (25, 27). This test was conducted to determine whether they were responders to GVS, as per previously conducted methodology (19).

Next, the vHIT was conducted in each nGVS condition: sham, 0.2, 0.6, and 1.2 mA. In one vHIT, passive head rotation to the right and left was performed until 20 successes were achieved in each direction (28) under all of the nGVS conditions. The nGVS was delivered at 70 s, and the vHIT was completed within 40 s during nGVS. The order of stimuli was randomized for all participants. After the vHIT, we measured the center of pressure (COP) of the feet while standing upright during the sham, 0.2, 0.6, and 1.2 mA nGVS conditions. Similarly, the order of the nGVS conditions was randomized for all participants. The interval between tests was approximately 30 s. In summary, four vHITs (40 s) and four COP measurements (30 s) were conducted under random stimulation conditions.

### vHIT

All vHITs (5) were administered by one tester, who is a skilled physical therapist specializing in otolaryngology. Left eye position and head velocity were recorded using the high-speed digital EyeSeeCam system (220 frames/s with an inertial measurement unit gyroscope; Interacoustics, Middelfart, Denmark) with infrared camera to estimate the gain of VOR, as per previously established methodology (29). During the test, participants wore tight-fitting goggles and were seated in a chair, instructed to make sure their pupils were clearly visible, and maintain their gaze on a red magnet (1 cm in diameter) attached to a whiteboard as a target positioned 1 m in front of them. The head was passively rotated by a tester at a velocity of more than 150°/s and a mean acceleration ranging from 1,000 to 2,500°/s^2^ (29). The horizontal rotation amplitude was set at 5–10°. Trials not meeting these conditions were omitted from the successful trials. Twenty suitable impulses of head rotational to the right and left were recorded in each stimulation condition, as previously described (29). Individual VOR gains were automatically calculated with the software included with EyeSeeCam system.

### COP Measurements

Participants were instructed to maintain an upright standing position on a force plate with both feet together while looking straight ahead to gaze at a blue magnet target (1 cm in diameter) 2 m in front of the participant. The ground reaction force during standing was recorded for 30 s with a force plate (Gravicorder G5500; Anima, Japan) at a sampling rate of 20 Hz. To estimate postural sway, the total path length of the foot center of pressure (COP-TL) during standing for 30 s was calculated as described in our previous studies (19, 25).

### nGVS

The nGVS experiments were conducted in nearly the same manner as previous studies (14, 18, 19, 30). To deliver the noise electrical stimulation to the vestibular nerve, we used DC-STIMULATOR PLUS (Eldith, NeuroConn GmbH, Ilmenau, Germany) via Ag/AgCl surface electrodes on both mastoid processes as per our previous studies (14, 19, 30). The “noise” stimulation mode was used to generate a current in random level generated for every sample; the sample rate was 1,280 samples/s (14, 18, 19, 30, 31). The intensity was set to 0.2, 0.6, and 1.2 mA in this “noise” mode. In this setting, random numbers are normally distributed over time. The probability density follows a Gaussian bell curve, and all the coefficients have a similar size in the frequency spectrum in this “noise” mode. For the sham condition, direct current electrical stimulation was delivered using the same device with the intensity set to 0 mA (control trial) as described previously (19). These stimulations were delivered for 70 s during the vHIT and 50 s during the COP measurements.

### Analysis

**Figure 1:**
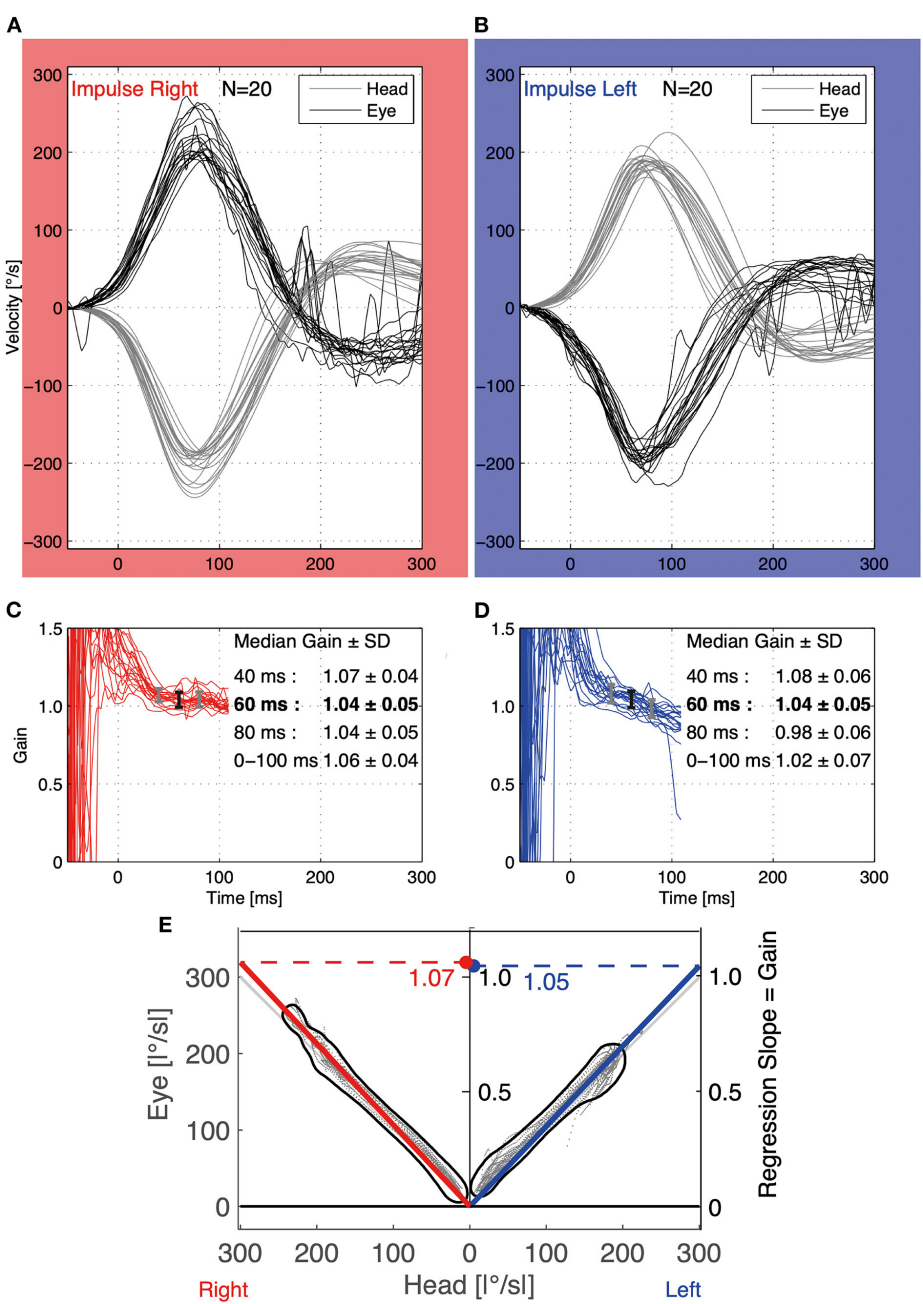
Representative waveform of eye and head velocity for head impulses to the right **(A)** and left **(B)** in a participant during the vHIT (20 head impulses to the right and left). The vertical and horizontal axes indicate velocity and time from the start of head motion (head motion velocity >20°/s), respectively. The gray lines indicate head velocity, while the black lines indicate eye velocity. In the middle graphs, the 20 red **(C)** and blue **(D)** waves indicate VOR-gain calculated from eye and head velocity in each impulse, respectively. The vertical and horizontal axes indicate VOR-gain and time from the start of head motion, respectively. The vertical bars indicate the median and standard deviation (SD) at 40 ms (left gray bar), at 60 ms (middle black bar), and at 80 ms (right gray bar) in **(C,D)**. The median VOR-gain at 60 ms was used for analysis as in each vHIT, as this value especially reflects the function of the horizontal canal during horizontal rotational HIT. **(E)** The bottom graph is a scatter plot of absolute eye and head velocity for head impulses to the right and left in the vHIT, and the red and blue lines represent the respective regression lines. The regression slope (RS) was used as VOR-gain-RS for analysis, as this indicates the ratio of eye/head velocity during the whole motion. VOR, vestibulo-ocular reflex; vHIT, video head impulse test.

**Figure 2:**
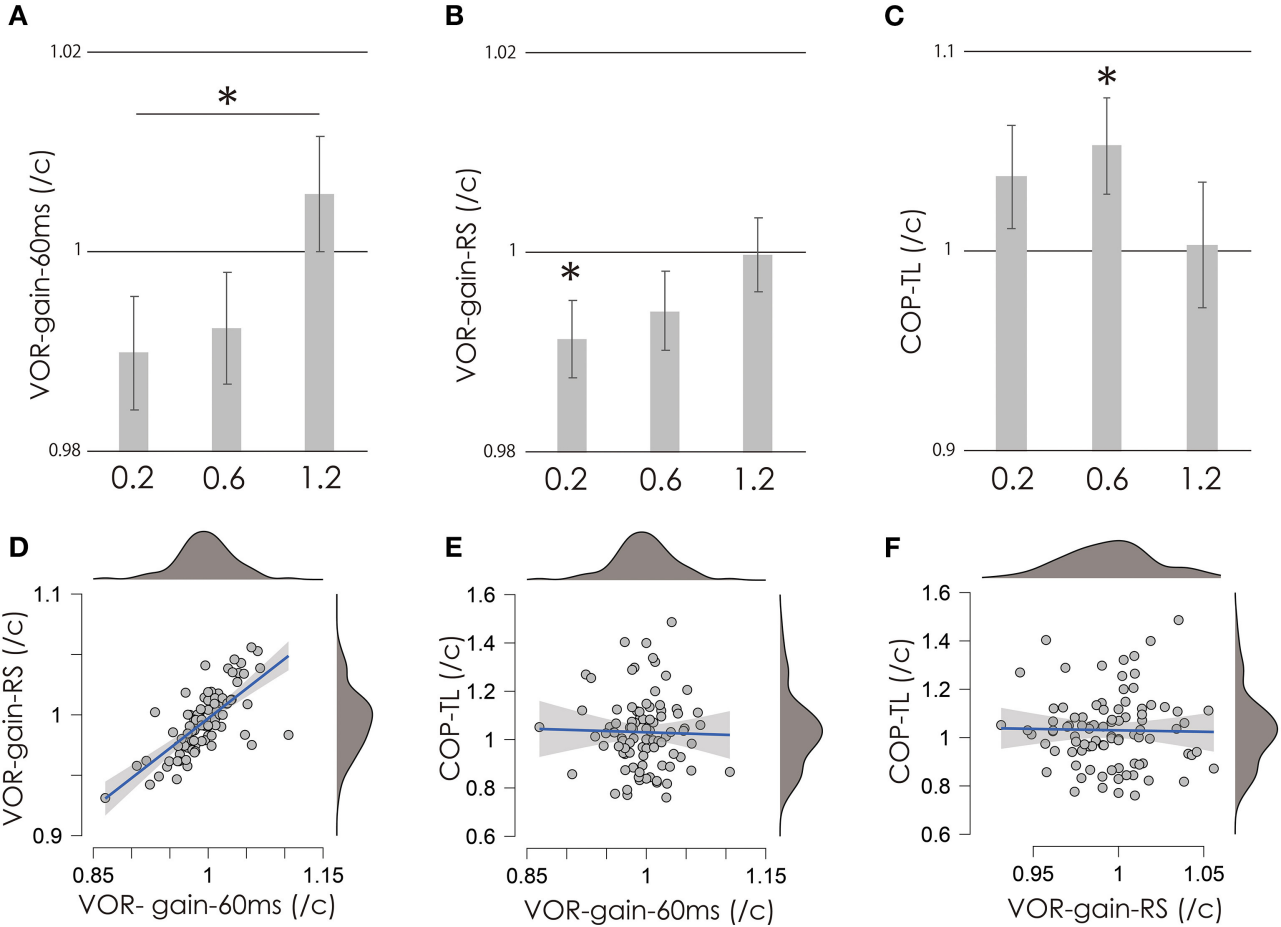
VOR-gain-60 ms **(A)**, VOR-gain-RS **(B)**, and COP-TL **(C)** per control ratio at 0.2, 0.6, and 1.2 mA. Vertical gray bars indicate the mean, and error bars indicate standard errors. Asterisks indicate significance. **(D–F)** Scatter plots of COP-TL, VOR-gain-60 ms, and VOR-gain-RS over all stimulation conditions. Small gray circles indicate individual data, while the mean value of the right and left VOR was used as a representative value (see Methods and Results sections). The blue lines indicate regression lines, and the gray area indicates the 95% confidence interval. Upper and right dark gray areas indicate the density of the data (top: 100%; bottom: 0%). VOR, vestibulo-ocular reflex; vHIT, video head impulse test; COP-TL, total path length of the foot center of pressure; RS, regression slope.

Figure 1 shows typical waveforms for eye and head velocity. Vestibulo-ocular reflex gain was calculated as eye motion velocity/head motion velocity at 40, 60, and 80 ms after the start of head motion by averaging the EyeSeeCam (Interacoustics, Middelfart, Denmark) results for 20 successful trials in each vHIT, as per previous methodology (29). The start of head motion was defined as the point when the velocity exceeded 20°/s, which is the default setting in the EyeSeeCam system and has been used previously (29). The VOR gain in 60 ms (VOR-gain-60 ms) is considered the most reflective parameter of the VOR function (32). The regression slope (RS) was calculated by determining the slope of the best-fitting line for the head and eye velocities (33) (Figure 2), which indicates the VOR gain in the whole eye and head (8). Therefore, we used the VOR gain in 60 ms and RS to estimate VOR function. Rotation to the right primarily reflects the function of the right horizontal semicircular canal, while rotation to the left primarily reflects the function of the left horizontal semicircular canal (5). Therefore, the VOR for the right and left impulses obtained from one participant were regarded as individual parameters (34). However, to estimate the correlation between the effect of nGVS on body sway (COP-TL) and VOR, the mean VOR for the right and left impulses was calculated as one parameter.

The total path length of the COP position (COP-TL) for 30 s was calculated to estimate the amount of body sway. The test/control ratio was calculated to normalize these parameters. A one-sample Student's *t*-test was performed to test the hypothesis that the test/control ratio differs from 1. However, if the normality test (Shapiro–Wilk) was significant, the Wilcoxon test was adopted. An OR-ANOVA was used to examine differences among intensities. If the Shapiro–Wilk test suggested an equal distribution for the ANOVA, the Friedman test was used. If the ANOVA suggested a significant effect of intensity, a *post-hoc* Bonferroni test or Kruskal–Wallis test was conducted. The JASP software (version 0.14.1; University of Amsterdam, Amsterdam, the Netherlands) (35) was used for all statistical analyses, and the alpha level was set to 0.05.

## Results

We made sure there were only the responders to GVS in this study because all participants induced lateral body sway to the anodal side by spGVS before the experiments. No participants experienced harmful side effects (i.e., headache, epilepsy, burns, or continuous dizziness after stimulation) throughout the entire experiment.

Figures 2A–C shows the test/control ratios for VOR-gain (60 ms), VOR-gain (RS), and COP-TL. Table 1 shows the results of the one-sample *t*-test, which revealed significant changes in VOR-gain (RS) and COP-TL from the control at 0.2 at 1.2 mA, respectively. Table 2 shows the results of the OR-ANOVA, which suggested a significant effect of stimulation on VOR-gain (60 ms), although there was no significant effect of stimulation on VOR-gain (RS) or COP-TL. The *post-hoc* analysis for VOR-gain (60 ms) revealed a significant difference between 0.2 and 1.2 mA (Table 3). Figure 2 shows a scatter plot of VOR-gain (60 ms), VOR-gain (RS), and COP-TL over the stimulation conditions. Table 4 shows the results of the correlation analysis for VOR-gain (60 ms), VOR-gain (RS), and COP-TL in the 0.2, 0.6, and 1.2 mA conditions and over all conditions. The results suggested that there was a significant correlation between VOR-gain (60 ms) and (RS), but not between VOR-gain (60 ms) and COP-TL.

**Table 1:** One-sample test.

		**Test of normality (Shapiro-Wilk)**							**95% CI for location estimate**		
**Parameter**	**Intensity (mA)**	***W***	***p***	**Test**	**Statistic**	***df***	***p***	**Location estimate**	**Lower**	**Upper**	**Significant**
VOR-gain-60 ms	0.2	0.96	0.049	Student	−1.793	59	0.078	0.99	0.978	1.001	
				Wilcoxon	573.5		0.147	0.991	0.979	1.002	
	0.6	0.991	0.924	Student	−1.387	59	0.171	0.992	0.981	1.003	
				Wilcoxon	607.5		0.175	0.991	0.979	1.003	
	1.2	0.972	0.189	Student	0.983	59	0.33	1.006	0.994	1.017	
				Wilcoxon	840.5		0.27	1.008	0.994	1.019	
VOR-gain-RS	0.2	0.948	0.013	Student	−2.261	59	0.027	0.991	0.984	0.999	
				Wilcoxon	353		0.026	0.988	0.979	0.997	^*^
	0.6	0.975	0.265	Student	−1.501	59	0.139	0.994	0.986	1.002	
				Wilcoxon	608		0.176	0.992	0.983	1.002	
	1.2	0.945	0.009	Student	−0.084	59	0.933	1	0.992	1.007	
				Wilcoxon	677		0.917	1	0.987	1.008	
COP-TL	0.2	0.983	0.887	Student	1.436	29	0.162	1.037	0.984	1.09	
				Wilcoxon	296		0.198	1.03	0.982	1.084	
	0.6	0.946	0.134	Student	2.177	29	0.038	1.052	1.003	1.102	^*^
				Wilcoxon	329		0.047	1.052	1	1.092	
	1.2	0.911	0.016	Student	0.088	29	0.931	1.003	0.938	1.068	
				Wilcoxon	201		0.529	0.983	0.928	1.047	

*For the Student t-test, location estimate is given by the sample mean and the alternative hypothesis specifies that the mean is different from 1. For the Wilcoxon test, location estimate is given by the Hodges-Lehmann estimate, and the alternative hypothesis specifies that the median is different from 1*.

* *p < 0.05*.

**Table 2:** ANOVA.

	**Cases**	**Sum of squares**	***df***	**Mean square**	***F***	***η^2^***	***p***	**Significant**
VOR-gain-60 ms	Intensity	0.009	2	0.004	3.564	0.057	0.031	^*^
	Residuals	0.144	118	0.001				
VOR-gain-RS	Intensity	0.002	2	0.001	2.209	0.036	0.114	
	Residuals	0.059	118	4.970e−4				
COP-TL	Intensity	0.039	2	0.019	1.882	0.061	0.161	
	Residuals	0.597	58	0.01				

* *p < 0.05*.

**Table 3:** *Post-hoc* test in VOR-gain-60 ms.

			**95% CI for mean difference**					
		**Mean difference**	**Lower**	**Upper**	***SE***	***t***	**p _**holm**_**	**Significant**
0.2 mA	0.6 mA	−0.002	−0.018	0.013	0.006	−0.384	0.702	
0.2 mA	1.2 mA	−0.016	−0.031	−3.297e−4	0.006	−2.48	0.044	^*^
0.6 mA	1.2 mA	−0.013	−0.029	0.002	0.006	−2.096	0.076	

*P-value and confidence intervals adjusted for comparing a family of three estimates (confidence intervals corrected using the Bonferroni method)*.

* *p < 0.05*.

**Table 4:** Correlation.

					**Pearson**			**Spearman**		
	**Parameter 1**	**Parameter 2**	**Shapiro-Wilk**	***p***	***r***	***p***	**Significant**	**Rho**	***p***	**Significant**
Total	VOR-gain-60 ms	VOR-gain-RS	0.883	<0.001	0.711	<0.001	^***^	0.712	<0.001	^***^
	VOR-gain-60 ms	COP-TL	0.98	0.167	−0.026	0.81		0.006	0.955	
	VOR-gain-RS	COP-TL	0.969	0.03	−0.021	0.848		0.002	0.986	
0.2mA	VOR-gain-60 ms	VOR-gain-RS	0.911	0.016	0.754	<0.001	^***^	0.669	<0.001	^***^
	VOR-gain-60 ms	COP-TL	0.905	0.011	−0.011	0.955		0.078	0.681	
	VOR-gain-RS	COP-TL	0.962	0.339	−0.249	0.184		−0.147	0.437	
0.6mA	VOR-gain-60 ms	VOR-gain-RS	0.924	0.034	0.677	<0.001	^***^	0.663	<0.001	^***^
	VOR-gain-60 ms	COP-TL	0.954	0.215	−0.096	0.614		−0.054	0.776	
	VOR-gain-RS	COP-TL	0.966	0.44	0.084	0.659		0.109	0.564	
1.2mA	VOR-gain-60 ms	VOR-gain-RS	0.71	<0.001	0.683	<0.001	^***^	0.829	<0.001	^***^
	VOR-gain-60 ms	COP-TL	0.954	0.215	0.077	0.685		0.073	0.702	
	VOR-gain-RS	COP-TL	0.933	0.059	0.124	0.513		0.112	0.554	

*** *p < 0.001*.

## Discussion

The present study investigated the effect of nGVS on the VOR in humans. Specifically, we investigated whether nGVS modulates the VOR-gain in 60 ms and RS, and whether nGVS is correlated with VOR and COP-TL. Our findings indicated that VOR-gain-RS was significantly reduced by nGVS at 0.2 mA, and that COP-TL was significantly increased by nGVS at 0.6 mA. We also observed a significant difference in VOR-gain-60 ms between the 0.2 and 1.2 mA conditions. However, there was no significant correlation between COP-TL and VOR-related parameters. These findings indicate that nGVS at 0.2 mA can inhibit the VOR, while nGVS at 0.6 mA can increase body sway while standing upright in healthy individuals, although there may be no relationship between the respective effects.

Administration of nGVS at 0.2 mA considerably reduced VOR-gain-RS, indicating that low-intensity nGVS inhibits the VOR. In contrast, VOR-gain-60 ms differed significantly between the 0.2 and 1.2 mA conditions, suggesting that high-intensity nGVS may increase the VOR. The vHIT explores Type I Hair Cells activity and consequently the afferent transient system (5). Ballistic rotation of the head in vHIT triggers action potentials in primary vestibular afferent neurons that project to vestibular nuclei, and induces eye movement (36). Galvanic vestibular stimulation can affect the synapses between hair cells in the semicircular canal and primary vestibular nerve, in addition to directly affecting the primary vestibular nerve including otolithic and semicircular canal neurons (11, 37). Furthermore, a previous *in vitro* study reported that stochastic noise electrical stimulation of the vestibular nuclei can modulate the neuronal gain of the medial vestibular nuclei (38). The nGVS can regulate the vestibular information carried by the vestibular neurons originating from Type I Hair Cells (16). Therefore, in this study, we propose that the nGVS modulated the VOR gain. However, the effect of nGVS is intensity-dependent; the optimal intensity decreases body sway, while non-optimal intensities increase body sway (20). Therefore, based on our results and these findings, we speculate that stimulation at 0.2 mA may be optimal for inhibition of the VOR, while that at 1.2 mA may be optimal for facilitation of the VOR.

The COP-TL was significantly increased by nGVS at 0.6 mA. A previous study reported that nGVS at 0.2 mA decreased body sway in patients with vestibular disorder, while that at 0.5 mA increased body sway (20). Another previous study similarly reported that nGVS at 1 mA increased the COP-TL in a healthy young population (19). Therefore, our finding that nGVS at 0.6 mA increases COP-TL is consistent with those of previous studies. On the other hand, no significant decreases in COP-TL were observed at any intensity in the present study. A previous study reported that the effect of nGVS on body sway depends on the amount of body sway without stimulation (18), suggesting that only individuals with balance impairments can benefit from nGVS. As our study included healthy young adults without any neurological disorders, this may explain why nGVS only induced increases in COP-TL.

There was a significant positive correlation between VOR-gain-60 ms/control and VOR-gain-RS/control (Table 4). Vestibulo-ocular reflex-gain-60 ms reflects the ratio of eye motion velocity/head motion velocity at 60 ms after the start of head rotation, while the test/control parameter reflects the effect of nGVS. On the other hand, VOR-gain-RS reflects the ratio of eye motion velocity/head motion velocity during movement. A previous study indicated that values at 60 ms especially reflect the function of the ipsilateral horizontal semicircular canal, while those at other points reflect other functions. For example, values at 40 ms reflect the function of the ipsilateral otolith, while those after 100 ms, including compensatory catch-up saccades (5), reflect cerebellar function (39). Therefore, VOR-gain-60 ms may specifically reflects the function of the semicircular canal, while RS may include both otolith and cerebellar function. The correlation of the effect on both suggests that nGVS may exert effects not only on the ipsilateral semicircular canal, but also on organs common to both the primary vestibular nerve and the vestibular nucleus.

On the other hand, we observed no significant correlation between COP-TL and any VOR-gain parameters in any stimulation condition (Table 4). This suggests that there is no relationship between the effect on VOR and that on body sway in healthy young individuals, reflecting the function of the vestibulospinal reflex. There are some possible reasons for this decorrelation: First, the optimal intensity for the VOR and body sway was not 0.2, 0.6, or 1.2 mA. Therefore, a more rigorous search for intensity may be necessary (e.g., in 0.5 mA increments). Next, nGVS affects VOR and body sway via different organs. The origin of the vestibulospinal response for postural control is considered to arise from vertical canal input and otolith input (40). On the other hand, the horizontal head impulse of the VOR is related to the horizontal semicircular canal (41). Further studies are required to investigate the effect of nGVS on the vertical VOR and body sway.

There were some limitations to this study. First, only healthy young individuals were included, meaning that the effects of nGVS on VOR and body sway observed in this study may not apply to older populations and patients with vestibular disorders, as these effects may depend on vestibular function (42). Further, we tested the response to GVS before the examination, and further studies are required to determine the optimal intensity (20) of nGVS for the VOR in patients with vestibular disorders or healthy older individuals. Lastly, we could not rigorously separate and remove predictionary or compensatory saccades from all eye movements. As the methods for identifying and separating these saccades remain inconclusive, further research is needed.

In conclusion, our findings indicated that nGVS can modulate VOR-gain. The effects of nGVS on VOR-gain may not be related to the effect on body sway during upright standing requiring vestibulospinal control. Further studies are required to determine the optimal intensity for improving VOR in patients with vestibular disorders.

## Data Availability Statement

The datasets presented in this study can be found in online repositories. The names of the repository and accession number can be found below: Akiyoshi Matsugi, Tomoyuki Shiozaki (2021), DATASET for vHIT-nGVS Mendeley Data, doi: 10.17632/dtj374wb2w.1, https://data.mendeley.com/datasets/dtj374wb2w/1.

## Ethics Statement

The studies involving human participants were reviewed and approved by Ethics Committee of Shijonawate Gakuen University. The patients/participants provided their written informed consent to participate in this study.

## Author Contributions

AM and TS: conceptualization and methodology. AM, TS, and HT: data curation, validation, writing—review, and editing. AM: formal analysis, funding acquisition, visualization, and writing—original draft. TS: resources and software. HT: supervision. All authors contributed to the article and approved the submitted version.

## Funding

This study was supported by the Japan Society for the Promotion of Science (JSPS) KAKENHI (grant number 20K11298) and by the Institute of Health Sciences in Shijonawate Gakuen University (grant number IHSS2002).

## Conflict of Interest

The authors declare that the research was conducted in the absence of any commercial or financial relationships that could be construed as a potential conflict of interest.

## Publisher's Note

All claims expressed in this article are solely those of the authors and do not necessarily represent those of their affiliated organizations, or those of the publisher, the editors and the reviewers. Any product that may be evaluated in this article, or claim that may be made by its manufacturer, is not guaranteed or endorsed by the publisher.
